# Evidence for Active Uptake and Deposition of Si-based Defenses in Tall Fescue

**DOI:** 10.3389/fpls.2017.01199

**Published:** 2017-07-18

**Authors:** Emma McLarnon, Simon McQueen-Mason, Ingo Lenk, Susan E. Hartley

**Affiliations:** ^1^Department of Biology, University of York York, United Kingdom; ^2^DLF Seeds Ltd. Hoejerupvej, Denmark

**Keywords:** silicon, inducible defense, *Festuca arundinacea*, stomatal conductance, transporter, tall fescue, stress

## Abstract

Silicon (Si) is taken up from the soil as monosilicic acid by plant roots, transported to leaves and deposited as phytoliths, amorphous silica (SiO_2_) bodies, which are a key component of anti-herbivore defense in grasses. Silicon transporters have been identified in many plant species, but the mechanisms underpinning Si transport remain poorly understood. Specifically, the extent to which Si uptake is a passive process, driven primarily by transpiration, or has both passive and active components remains disputed. Increases in foliar Si concentration following herbivory suggest plants may exercise some control over Si uptake and distribution. In order to investigate passive and active controls on Si accumulation, we examined both genetic and environmental influences on Si accumulation in the forage grass *Festuca arundinacea.* We studied three *F. arundinacea* varieties that differ in the levels of Si they accumulate. Varieties not only differed in Si concentration, but also in increases in Si accumulation in response to leaf damage. The varietal differences in Si concentration generally reflected differences in stomatal density and stomatal conductance, suggesting passive, transpiration-mediated mechanisms underpin these differences. Bagging plants after damage was employed to minimize differences in stomatal conductance between varieties and in response to damage. This treatment eliminated constitutive differences in leaf Si levels, but did not impair the damage-induced increases in Si uptake: damaged, bagged plants still had more leaf Si than undamaged, bagged plants in all three varieties. Preliminary differential gene expression analysis revealed that the active Si transporter Lsi2 was highly expressed in damaged unbagged plants compared with undamaged unbagged plants, suggesting damage-induced Si defenses are regulated at gene level. Our findings suggest that although differences in transpiration may be partially responsible for varietal differences in Si uptake, they cannot explain damage-induced increases in Si uptake and deposition, suggesting that wounding causes changes in Si uptake, distribution and deposition that likely involve active processes and changes in gene expression.

## Introduction

Silicon (Si) is considered a non-essential element, but it has many useful functions in plants (Guntzer et al., 2012). Plants take up Si in the form of monosilicic acid [Si(OH)_4_] via the roots (Ma et al., 2006). It is transported through the xylem and deposited in the leaves to form phytoliths. Phytoliths are solid bodies of silica (SiO_2_) found in epidermal layers, both within and between the plant cells (Piperno, 1988; Currie and Perry, 2007). Trichomes (small hairs found on the leaf surface) may also become enriched with Si and increase the abrasiveness of leaf surfaces. Plants within the grass family (Poaceae) accumulate Si in varying concentrations (up to 10% dry weight) where its primary function is to defend the leaf surface against a range of stresses including drought (Emam et al., 2014; Mitani-Ueno et al., 2016), pathogen attack (Fauteux et al., 2005; Liang et al., 2015) and herbivory (Massey et al., 2006, 2007; Hartley et al., 2015). Many species of grass show diversity in their reported shoot Si concentrations (Ma et al., 2001; Hodson et al., 2005; Massey and Hartley, 2006; Hunt et al., 2008). Differences in the density and efficiency of Si transporters may underpin these differences, as reported in rice (Wu et al., 2006; Ma et al., 2007), whilst environmental conditions such as water availability and herbivory can also drive changes in Si concentration (Quigley and Anderson, 2014; Wieczorek et al., 2015). The relative importance of genotypic, phenotypic and environmental factors for Si uptake remains unclear (Hartley et al., 2015; Hartley and DeGabriel, 2016).

Lsi1 is a root-specific Si transporter involved in the transport of Si from the soil solution [as Si(OH)_4_] to within the root, first identified in rice (Ma et al., 2006), though orthologues of Lsi1 have now been identified in other crop species (e.g., *Zea mays* L, Mitani et al., 2009a,b; *Hordeum vulgare* L., Chiba et al., 2009; Yamaji et al., 2012 and *Glycine max* (L) Merr., Deshmukh et al., 2013). Lsi1 in rice is a passive aquaporin-like transmembrane protein (Yamaji and Ma, 2007) which transports Si into the root cells, whilst a Si efflux transporter, Lsi2, actively pumps (driven by a proton gradient) Si out of the root cells and into the stele (Ma et al., 2007; Deshmukh and Bélanger, 2016). Aquaporins permit the passage of water through the cell membrane following the gradient in water potential, suggesting that Si can enter the plant cells without the need and use of Si specific transporters (Exley, 2015). Contrary to this, some studies have found that the Si transporters in rice (a hyper-Si accumulator, accumulating up to 10% Si in dry weight) and maize are down-regulated after constitutive Si supply (Yamaji and Ma, 2007; Mitani-Ueno et al., 2016), which would not be the case if the transport was purely via water flow into the cells. Furthermore, some studies have reported tissue Si concentrations above that plausible for passive transport only (Faisal et al., 2012; Yamaji and Ma, 2014; Yamaji et al., 2015). Si has been identified in plant parts with low transpiration such as the husk, presumably actively redirected to these locations by Si-mediated transporters (Yamaji and Ma, 2014). Silicon concentrations within specific plant tissues are not always strongly related with transpiration rate, with silicification of silica cells (specific epidermal cells filled with silica) mainly occurring at night (Blackman, 1969) when transpiration rates are low. Silicon deposition has also been found to be independent of water evapotranspiration (Kumar et al., 2016), even when transpiration played a role in the uptake of Si into plants. Further, evidence of silicification of live cells in the absence of transpiration suggests that the cells are actively moving Si into the cells independent of transpiration (Kumar et al., 2016). This may explain the highly organized and distinctive patterns of deposition observed in different species (Hartley et al., 2015).

Increases in Si uptake and changes in Si deposition in response to herbivory may also suggest active redirection of Si within the plant (Hartley et al., 2015). Silicon defenses are now known to be inducible, with up to 400% increases in Si in response to leaf damage (Massey and Hartley, 2006; Massey et al., 2007). Herbivory-induced increases in Si occur in response to a range of herbivores, persist for several months and have been demonstrated in the field (Massey and Hartley, 2006; Massey et al., 2007; Garbuzov et al., 2011; Reynolds et al., 2012; Soininen et al., 2013; Hartley et al., 2015; Wieczorek et al., 2015). To date, no studies have tested whether this increase occurs due to leaf damage leading to higher rates of water loss (i.e., increases in transpiration) and thus subsequent changes in uptake and deposition of Si, or if there is an up-regulation in Si transporter genes in the root, brought on by a damage response from the leaves.

*Festuca arundinacea* Schreb. (tall fescue) has been classified as both a Si accumulator (Hodson et al., 2005) and a non-accumulator (Ma et al., 2001), suggesting its Si uptake in the natural environment is not uniform. Silicon uptake and deposition is relatively uncharacterized within this species, though previous work (Hartley et al., 2015) has shown it has the ability to take up and deposit Si upon the leaf epidermis, and that the levels of Si within the leaf tissues and the structures it enriches differ amongst breeding varieties within the species (very very soft = 0.43%–0.69% Si, harsh = 0.46%–0.80% Si). Varieties have been described as harsh and soft in terms of their leaf texture, which reflects Si deposition (Hartley et al., 2015). However, how these varieties respond to damage, in terms of Si uptake, and whether any damage-induced increases in Si result from changes in passive Si uptake via transpiration or other more active processes mediated by plant defense responses has not been tested. To date, the studies that have investigated the effects of transpiration on Si uptake have not included an assessment of the effects of damage. Previous studies have focused on the role of transpiration in undamaged plants in cucumber over a short period of time (Faisal et al., 2012) or in detached leaves placed on solution to understand the silicification of cells within the leaf (Kumar et al., 2016). In contrast, our study investigates the effects of herbivore-simulated damage, in an attempt to understand mechanisms driving the induction process. The aim of this study was to determine if damage-induced increases in Si uptake could be explained by environmental variables such as differences in transpiration rates, or if Si-induced defenses are mediated at gene level by changes in Si transporter expression.

This study investigates how altering transpiration rate and simulating herbivory affects the Si concentration of three varieties of *F. arundinacea.* We hypothesize that:

(1) If Si uptake is largely a passive process associated primarily with transpiration rate, varietal differences in Si concentration will be driven by differences in stomatal conductance and stomatal density;
(2) Damage will induce an increase in Si uptake and varieties with a greater rate of Si uptake and deposition will also show a larger induction response and an increased expression of Si transporters;
(3) If damage-induced increases in Si uptake are driven by changes in water relations, then reducing transpiration differences between undamaged and damaged plants will prevent this increase in Si uptake after damage.

## Materials and Methods

### Plant Growth and Experimental Treatments

Three genotypically distinct breeding varieties of *F. arundinacea* contrasting in their ability to accumulate Si (under standard greenhouse conditions, average leaf Si concentrations: very very soft = 0.44%; very soft = 0.43%; and harsh = 0.55%) and varying in leaf texture were provided by the commercial seed company DLF Seeds Ltd., Denmark. The leaf texture is a qualitative trait measured and defined by plant breeders according to how harsh or soft the leaf texture felt on a numerical scale. These were:

- VVS (very very soft leaf texture);
- VS (very soft leaf texture);
- H (harsh leaf texture).

Plants were grown individually in a loam-based compost (John Innes No.2) in 13-cm plastic pots in standard greenhouse conditions: 16 h daylight, 20°C day, 15°C night. Once established, plants were randomly subjected to a combination of bagging and damage treatments:

- Undamaged;
- Damaged;
- Undamaged or damaged, then placed in perforated plastic bags.

The aim of the bagging treatment was to control water flow through the plant; bagging the plants would subject both damaged and undamaged plants to similar levels humidity, thus reducing transpiration (Sellin et al., 2014). Treatments were applied four weeks after sowing, with plants harvested 8 weeks later. There were ten replicate plants of each variety per treatment. Plants were watered twice a week with 100ml of deionized water with 150 mgL^-1^ dissolved sodium metasilicate (Na_2_SiO_3_⋅9H_2_O); tap water was added as required. In the treatments where damage was applied, half of the total leaves of each plant were damaged twice a week using a metal file. Damaged and undamaged leaves were separated at harvest and leaf Si concentration analyzed separately.

### Epidermal Peel Analysis

During the plant harvest, 5 cm of one leaf from eight replicate plants of each variety per treatment were clipped and painted with clear nail varnish. Transparent sticky tape was placed onto the nail varnish once dried, peeled off and the tape stuck to microscope slides. The slides were analyzed via Nikon Eclipse Ni-U light microscope (Nikon Instruments, Kingston Upon Thames, Surrey) for stomatal, trichome, and phytolith counts.

### Si Analysis by Portable X-Ray Fluorescence Spectrometry (P-XRF)

Si was analyzed by portable P-XRF, calibrated using Si-spiked synthetic methyl cellulose and validated using Certified Reference Materials of NCS DC73349 ‘Bush branches and leaves’ obtained from China National Analysis Center for Iron and Steel. Leaf material was ball milled (Retsch MM 400, Haan, Germany) for 2 min at a vibrational frequency of 30 Hz (60 min^-1^) with 2 cm diameter steel grinding balls in 25 ml grinding jars. Leaf material was pressed at 10 tons into 13 mm diameter pellets with a manual hydraulic press using a 13 mm die (Specac, Orpington, United Kingdom). Si analysis (% Si DW) was performed using a commercial P-XRF instrument (Niton XL3t900 GOLDD analyzer: Thermo Scientific Winchester, United Kingdom) held in a test stand (SmartStand, Thermo Scientific, Winchester, United Kingdom; Reidinger et al., 2012).

### Stomatal Conductance Measurements

Stomatal conductance measurements were taken using the Delta –t AP4-UM-3 porometer (Delta-T devices Ltd, Cambridge, United Kingdom). The porometer was calibrated according to the manufacturer’s instructions and then the porometer probe was placed on the leaf and the time taken for the leaf to release sufficient water vapor to change the relative humidity in a small chamber by a fixed amount was measured; once stabilized (i.e., the same value was observed for two consecutive readings), the stomatal conductance value was recorded. Five readings per variety, per treatment were taken 1 or 2 days after treatments on five different days. Separate readings of undamaged leaves and damaged leaves of damaged plants were taken.

### RNAseq and Differential Gene Expression Analysis (DGEA)

At harvest, three biological replicate samples of unbagged, undamaged, and damaged roots for the VVS and H varieties were flash frozen in liquid nitrogen for RNA extraction. RNA was extracted using TRIzol^TM^ Reagent method from 100 mg of root material according to manufacturer’s instructions (Invitrogen, United Kingdom). The RNA quality was checked on a 1% agarose gel to test for degradation and quantified using NanoDrop. DNA digestion and cDNA libraries were prepared and sequenced by Leeds Institute of Molecular Medicine (Leeds, United Kingdom). Sequencing was performed using Illumina HiSeq 3000 (Illumina, Inc., United States) using one lane for all libraries, comprising 2 × 150 bp paired end reads. For library assembly, low quality reads and adapter sequences were removed from the raw FASTQ files using Cutadapt^1^ with parameters set to: quality >20 and read length >75 bp. The transcriptome was assembled *de novo* using Trinity RNA-Seq2.1.1 according to the online user-guide^2^. Library reads were aligned to the transcriptome using bowtie2 (Langmead and Salzberg, 2012) and transcript abundance calculated using the RNA-Seq by Expectation Maximization (RSEM) method (Li and Dewey, 2011). Transcripts were annotated in Trinotate v3.0 using BLAST searches (*E* value < 10^-20^) against Swissprot. DGEA was carried out on the annotated transcripts using the edgeR package (Robinson et al., 2010; McCarthy et al., 2012) to test for differences in log fold changes (logFC) > 1 with a false discovery rate (FDR) set to <0.05 to correct *P*-values for multiple testing. To confirm the identity of Lsi2 sequences, the transcripts were searched for sequence similarity to using BLAST and their transmembrane domains were compared to the barley Lsi2 (accession AB447483.1; Mitani et al., 2009a) sequence using TMHMM Server v2.0^3^. The sequences for *Lsi2* (Supplementary Table S1) were only partial sequences, but the transmembrane domains found in these sequences closely matched those in the barley Lsi2 transporter.

### Statistical Analyses

All statistical analyses were performed using R (version 3.3.2). Analysis of variance (ANOVA) tests were used to test the main and interactive effects of variety, bagging and damage (using damaged leaves of damaged plants) on leaf Si concentration and stomatal conductance. Paired *t*-tests were used to test for statistical differences between undamaged leaves and damaged leaves of the same damaged plants, where the aim was to test for localized and systemic responses in Si uptake and differences in stomatal conductance. Bonferroni’s correction was applied for *t-*tests, setting the level of significance to *P* < 0.02. Generalized linear models were used to test the main effects of variety on stomatal, trichome, and phytolith densities. Linear models were used to check for normality and homogeneity of variance following Crawley (2007). Si (%) values were transformed using the arcsine square root transformation to meet the assumptions of the tests. Significance was set at *P* < 0.05 for all analyses other than *t*-tests. Linear regression was used to test for relationships between stomatal conductance and Si concentration. *Post hoc* Tukey tests were carried out and significance was set at *P* < 0.05. Where models did not meet the assumptions, generalized linear models were applied instead of linear models. Packages used for analyses were as follows: lsmeans package (Lenth, 2016), multcompView (Graves et al., 2015), and ggplot function from ggplot2 package (Wickham, 2009).

## Results

### Undamaged Plants

**FIGURE 1:**
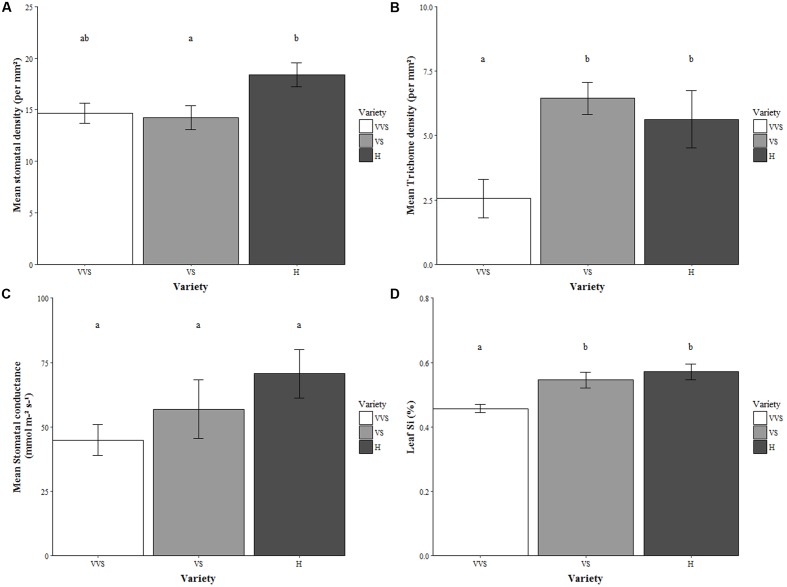
**(A)** Stomatal density, **(B)** Trichome density, **(C)** Stomatal conductance, and **(D)** Leaf Si concentration. VVS = very very soft, VS = very soft, and H = harsh. Values represent unbagged and undamaged plants. Bars are mean values ± SE. *N* = 9 for stomatal density, *n* = 5 stomatal conductance and *n* = 10 for leaf Si. Letters within bars denote significant differences between treatments (*post hoc* Tukey *p* < 0.05).

Stomatal conductance did not differ significantly between the three varieties but there was a trend for increased stomatal conductance with increasing harshness: VVS displayed the lowest stomatal conductance. Stomatal density, trichome density and Si concentration differed between variety. Stomatal density was higher in the H variety (*F*_2,23_ = 4.05, *P* = 0.03, **Figure 1A**) compared with the VS variety.

The VS and H varieties had more trichomes per mm^2^ compared to the VVS variety (*F*_2,23_ = 6.02, *P* = 0.008; **Figure 1B**), but phytolith density did not differ between the varieties (Supplementary Table S2).

**FIGURE 2:**
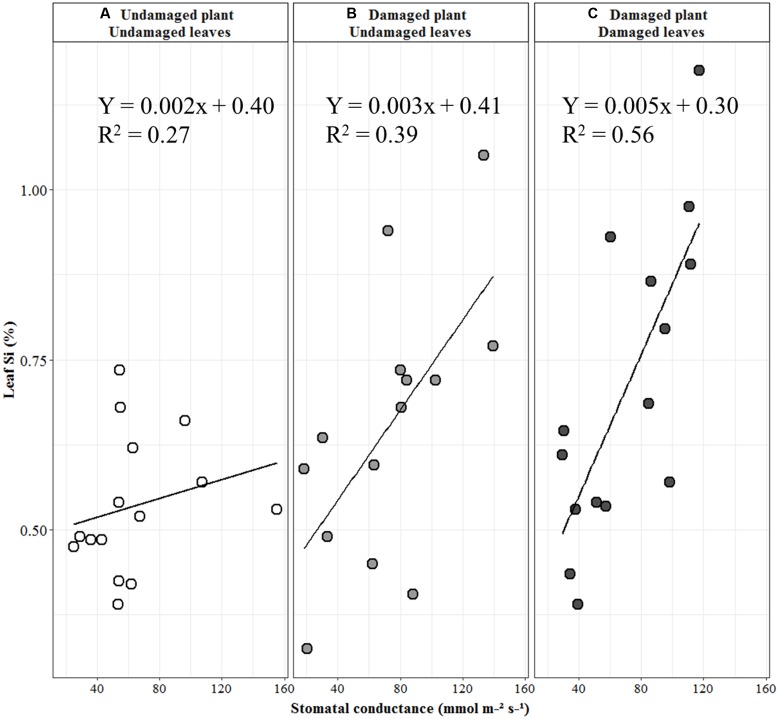
Linear regression between stomatal conductance and leaf Si concentration of unbagged plants. **(A)** Undamaged plants. **(B)** Damaged plants, undamaged leaves. **(C)** Damaged plants, damaged leaves. Regression line equation based on raw Si and stomatal conductance data; statistical analysis based on arcsine transformed Si data (see text for details).

The H and VVS varieties differed in their leaf Si concentration (*F*_2,18_ = 8.75, *P* = 0.002; **Figure 1D**). There was a positive relationship between stomatal conductance and Si concentration (*n* = 15, *r* = 0.52, *P* = 0.049; **Figure 2A**) in undamaged, unbagged plants.

The H variety had a higher expression of two *Lsi2* gene isoforms compared to the VVS variety in undamaged conditions (log fold changes = 3.72 and 7.60, FDR < 0.05, TRINITY_DN45085_c2_g1_i1 and TRINITY_DN45085_c2_g2_i2 in Supplementary Table S1).

### Damaged Plants

**FIGURE 3:**
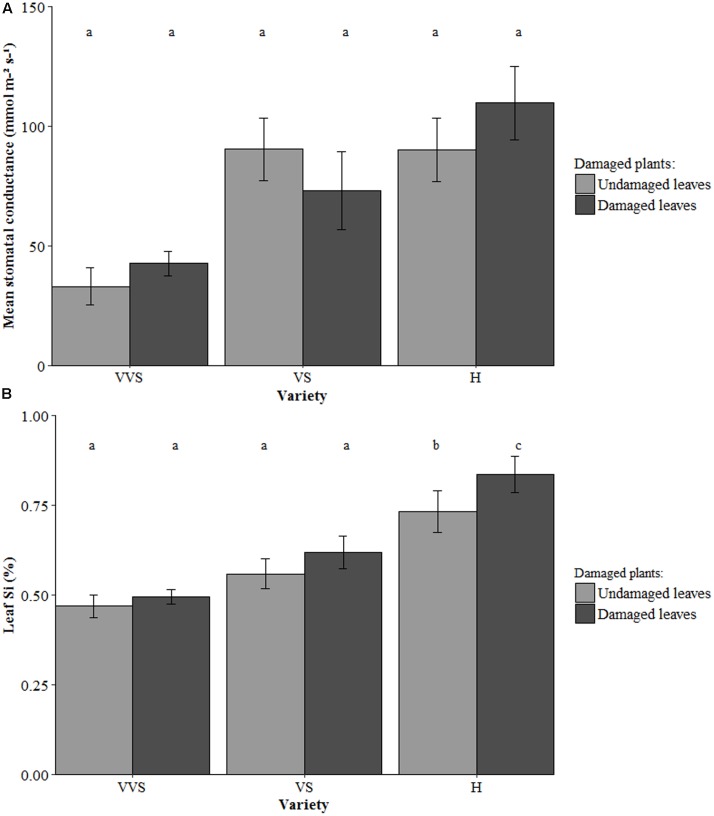
**(A)** Stomatal conductance, and **(B)** Leaf Si concentration of damaged plants in unbagged conditions. VVS = very very soft, VS = very soft and H = harsh. Bars are mean values ± SE. *N* = 5 for stomatal conductance and *n* = 10 for Si concentration. Letters within bars denote significant differences between treatments (*post hoc* Tukey *p* < 0.05).

Stomatal conductance was higher in the damaged leaves of damaged plants in the H variety compared with the VVS variety (*F*_2,12_ = 6.38, *P* = 0.01; **Figure 3A**). There were no differences in stomatal conductance between undamaged leaves and damaged leaves of damaged plants in any of the three varieties.

In undamaged leaves of damaged plants, the VVS variety had significantly fewer trichomes per mm^2^ compared to the VS and H variety (*F*_2,23_ = 5.03, *P* = 0.02; Supplementary Table S2). In the damaged leaves of damaged plants, no significant varietal differences were observed in terms of trichome density – although there was still a trend for the VVS variety to have fewer trichomes compared to the VS and H variety. Phytolith density was highest in the VS variety for both undamaged leaves of damaged plants (*F*_2,23_ = 8.20, *P* = 0.002) and damaged leaves of damaged plants (*F*_2,23_ = 813.83, *P* < 0.001; Supplementary Table S2).

The damaged leaves of damaged H plants had more Si than both VS and VVS subjected to this treatment (*F*_2,27_ = 19.89, *P* < 0.001; **Figure 3B**). Paired *t*-tests between undamaged leaves and damaged leaves of damaged plants showed there was a localized response to Si uptake in the H variety only – i.e., the damaged leaves had more Si compared to the undamaged leaves of the same plant (*t* = 4.58, *df* = 8, *P* = 0.002). There was a significant positive linear relationship between leaf Si concentration and stomatal conductance under damaged, unbagged conditions for both undamaged leaves (*n* = 15, *r* = 0.62, *P* = 0.02; **Figure 2B**) and damaged leaves of damaged plants (*n* = 15, *r* = 0.75, *P* = 0.001; **Figure 2C**).

In unbagged, damaged conditions showed that three *Lsi2* gene isoforms were expressed, and these were up-regulated in the H variety compared to the VVS variety (log fold changes = 4.52, 3.51 and 6.78, FDR < 0.05, TRINITY_DN45085_c1_g1_i1, TRINITY_DN45085_c2_g1_i1 and TRINITY_DN45085_c2_g2_i2 in Supplementary Table S1).

### Bagged Plants

Under bagged conditions, the patterns of stomatal conductance between varieties were similar to those in unbagged conditions. VVS had significantly lower stomatal conductance compared to VS and H varieties (*F*_2,24_ = 19.07, *P* < 0.001; data not shown).

The VVS variety had significantly fewer trichomes compared to the VS and H varieties under undamaged, bagged conditions (*F*_2,22_ = 10.96, *P* < 0.001). This relationship was the same for both undamaged leaves (*F*_2,22_ = 10.07, *P* < 0.001, Supplementary Table S3) and damaged leaves of damaged plants (*F*_2,22_ = 6.39, *P* = 0.007, Supplementary Table S3). Phytolith density was higher in the VS variety compared with H and VVS in undamaged, bagged plants (*F*_2,22_ = 8.63, *P* = 0.002, Supplementary Table S3) and also in damaged, bagged plants with undamaged leaves (*F*_2,22_ = 12.43, *P* < 0.001, Supplementary Table S3). The H variety had significantly fewer phytoliths on damaged leaves compared with the other two varieties under damaged, bagged conditions (*F*_2,22_ = 6.23, *P* = 0.007, Supplementary Table S3).

**FIGURE 4:**
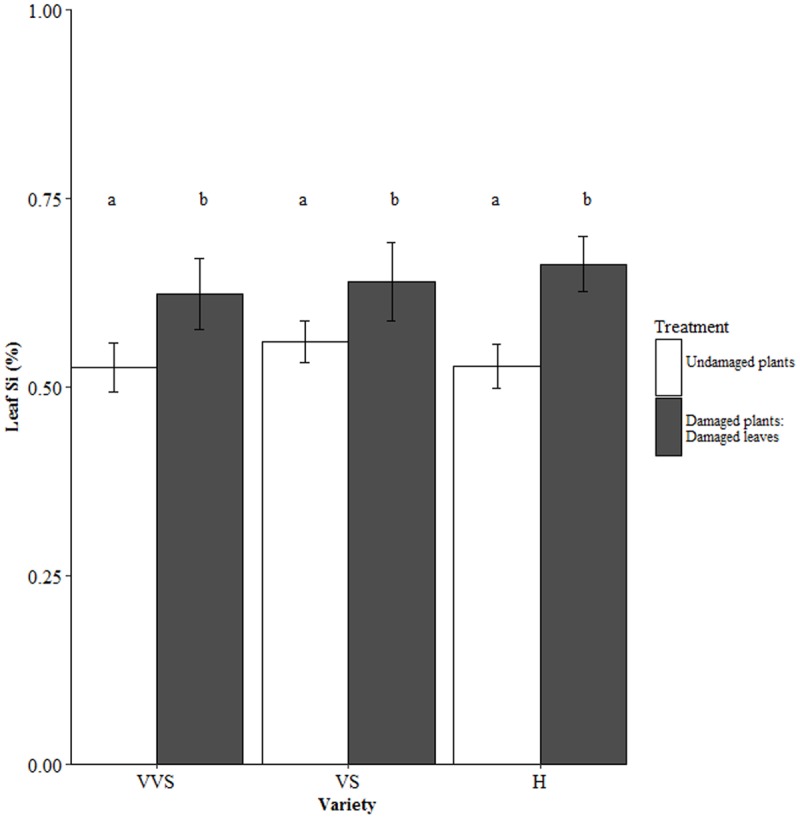
Leaf Si concentration of undamaged plants and damaged leaves of damaged plants under bagged conditions. VVS = very very soft, VS = very soft and H = harsh. Bars are mean values ± SE. *N* = 10. Letters within bars denote significant differences between treatments (*post hoc* Tukey *p* < 0.05).

In bagged conditions, leaf Si concentration did not differ between varieties in either the damaged leaves or undamaged leaves (**Figure 4**). However, damaged leaves of damaged plants had significantly higher leaf Si than undamaged plants in all three varieties (*F*_1,54_ = 11.21, *P* = 0.001; **Figure 4**). No relationship between leaf Si concentration and stomatal conductance was reported for undamaged, bagged plants or for damaged leaves of damaged, bagged plants. There was a weak relationship between leaf Si concentration and stomatal conductance in the undamaged leaves of damaged, bagged plants (*F*_1,13_ = 0.33, *P* = 0.03).

## Discussion

There are clear differences in the accumulation and deposition of Si between the varieties, and in how the varieties respond to damage in terms of induction of Si defenses. In unbagged conditions, these varietal differences tend to reflect similar differences in stomatal density and stomatal conductance, with the H variety tending to have the highest Si concentration and trichome density as well as the highest stomatal density and stomatal conductance, and with VVS having the lowest. Silicon concentration is significantly positively correlated with stomatal conductance in these plants. The H variety also shows higher induction of Si uptake after damage than the two soft varieties, increased expression of the active Si transporter *Lsi2* and shows some evidence of systemic induction the other two varieties do not show. However, in bagged conditions, these varietal differences disappear – undamaged bagged plants have the same Si concentration, regardless of variety, and all varieties respond to damage with induction in Si defenses and we no longer see the systemic induction in H plants. Further, these varieties continue to deposit trichomes and phytoliths on the leaf surface under bagged conditions in similar quantities to the unbagged plants, despite the likely difference in transpiration between these two conditions. These findings cannot be explained purely by passive processes linked to water evapotranspiration, implying that damage-inducted increases in Si deposition require active physiologically regulated processes (Kumar et al., 2016).

### Undamaged Plants

We hypothesized that if Si uptake was largely a passive process associated with transpiration rate, varietal differences in Si concentration would be driven by differences in stomatal conductance and stomatal density because Si uptake into the tissues, although mediated by the Si transporters, mainly follows the flow of water from the external environment into the root cells (Raven, 1983; Epstein, 1994, 1999; Exley, 2015). Our findings of a correlation between stomatal conductance and Si support this hypothesis of a strong role of the transpiration stream in Si uptake also found in previous studies (Sangster and Parry, 1971; Henriet et al., 2006; Cornelis et al., 2010; Faisal et al., 2012; Kumar et al., 2016) and suggests a strong role of the transpiration stream in Si uptake. However, the clear differences in Si concentration between the varieties, despite no statistical differences observed in stomatal conductance between them, suggests other factors than transpiration stream may have some influence on Si accumulation and deposition in the undamaged plants. The H variety had a higher expression of the active Si transporter *Lsi2* compared to the VVS variety; varietal differences have also been reported in barley cultivars in expression of *Lsi2*, where Si concentration was positively correlated with *Lsi2* expression (Mitani et al., 2009a). It was reported that constant Si supply led to the down-regulation of *Lsi2* in barley and maize over a period of a week (Mitani et al., 2009a). In our study, Si was constantly supplied over a period of 12 weeks, and it is possible that the VVS variety is less able to upregulate *Lsi2* than the H variety under these conditions. These results suggest Lsi2 has an important role in driving varietal differences in terms of Si concentration in tall fescue.

### Damaged Plants

We hypothesized that damaging plants would induce an increase in Si uptake, and that varieties with a greater rate of Si uptake and deposition would show larger induction responses. However, in unbagged conditions damaging leaves only elicited a response from the H variety, both systemically and locally. The undamaged leaves of damaged plants increased leaf Si concentration by 27% and the damaged leaves by 47% compared to the undamaged plants. Such increases in Si after induction have been observed in many other studies (Massey and Hartley, 2006; Massey et al., 2007; Garbuzov et al., 2011; Reynolds et al., 2012; Soininen et al., 2013; Hartley et al., 2015). Although under undamaged conditions varieties did not differ significantly in stomatal conductance (though there was a trend for higher conductance in harsher varieties), in damaged plants the H variety had significantly higher stomatal conductance than the VVS and VS varieties (see **Figures 1C**, **3A**). This suggests that varietal differences in Si in damaged, unbagged plants may at least in part, be driven by the uptake of water. The lack of response in stomatal conductance, by the VS and VVS varieties is surprising given that most studies (Warrington et al., 1989; Oleksyn et al., 1998; Aldea et al., 2005; Pincebourde et al., 2006) find an increase in stomatal conductance and transpiration when leaves are grazed or perforated, due to damage of the stomata causing impaired function, such as altering the ability of the guard cells to open and close properly. There was also a lack of response to damage in terms of increased Si uptake by VVS and VS varieties, but the VS variety had more phytoliths per mm^2^ than in undamaged plants. Thus, although Si concentration did not increase, this variety invested more Si in phytolith production suggesting a shift in allocation patterns of Si under damaged conditions. In damaged plants, there was a greater expression of *Lsi2* gene isoforms compared to the undamaged plants suggesting that this transporter is at least partially responsible for Si-induced defenses in this species. The *Lsi2* transporters were up-regulated in the H variety compared to the VVS variety. Tall fescue is an outbreeding, allohexaploid (Gibson and Newman, 2001) and therefore there may be splice variants of these Si transporters in the different varieties which are only activated upon damage. We were able to see differences between treatments using a small number biological replicates in a species with a complex genome such as tall fescue, providing clear evidence that Si-induced defenses are under molecular control in this species. In barley, Si concentration was positively correlated with *Lsi2* expression (Mitani et al., 2009a), here we also see plants with more Si in the leaves also have a higher expression of *Lsi2*.

### Bagged, Undamaged, and Damaged Plants

We hypothesized that if damage induced increases in Si uptake were driven by changes in water relations, bagging plants would prevent this increase in Si uptake after damage. Bagging the plants removed the differences observed between the undamaged and damaged plants in terms of stomatal conductance compared to when plants were not bagged, and also removed the varietal Si differences observed in unbagged plants. However, bagging plants did not remove the Si differences between the undamaged and damaged plants: there was still an induction response to damage, increasing the leaf Si concentration in damaged plants compared to the undamaged plants in all 3 varieties. The systemic induction in the H variety observed in unbagged damaged plants was not found in this treatment, suggesting systemic induction is in part influenced by water relations, but localized responses to damage with increased Si deposition are not. The trend in trichome and phytolith deposition between the varieties remains similar between unbagged and bagged conditions (i.e., that VVS has less trichomes compared to the VS and H variety and that the VS variety has more phytoliths compared with the H and VVS varieties), again suggesting this deposition is not primarily transpiration driven. We also see differences between the varieties in terms of the deposition patterns, even though the stomatal conductance is the same (Supplementary Table S3). Transpiration seems necessary for plants to accumulate Si from the roots to the leaf tissues, but other active means must be at play during the deposition to explain findings in our study. Other work supports this assertion, silica accumulation in silica cells takes place only during leaf development (Sangster, 1970; Motomura et al., 2006; Kumar et al., 2016); if transpiration were the sole cause of Si deposition then all leaves (despite their age) would continue to deposit Si in the silica cells, but this is not the case (Kumar et al., 2016).

Studies that have investigated the relationship between passive/ active uptake of Si in plants have found content of Si both higher (Faisal et al., 2012; Gocke et al., 2013; Kumar et al., 2016) and lower Si than expected for passive uptake (Cornelis et al.2010), which again goes against the suggestion that Si uptake and distribution is a purely passive process (Exley, 2015). Silicic acid may move freely into the roots but uptake and distribution of Si increases in the presence of the influx and efflux transporters (Ma and Yamaji, 2008; Farooq and Dietz, 2015). Many studies have shown Si transporters are responsible for the uptake and distribution of Si in different grass species (Ma et al., 2006; Chiba et al., 2009; Mitani et al., 2009a,b; Montpetit et al., 2012). Si transport, both within and between species is variable as is the regulation of the Si transporters – *Lsi1* is down regulated in rice during constant Si supply after 3 days (Ma et al., 2006) whereas in barley and maize for example, the expression is constitutive (Chiba et al., 2009; Mitani et al., 2009a). In terms of inducible plant defenses, plants may only up-regulate expression of Si transporters as needed and rely on their base transcript levels of Si transporters and transpiration to utilize Si under undamaged, unbagged conditions. Complex interactions between genetic and environmental controls on the expression of transporters may explain why Si levels for the same species are often so variable (Ma et al., 2001; Hodson et al., 2005; Soininen et al., 2013).

Given that Si transporters have been identified in many other species of grass such as rice (Ma et al., 2006) and barley (Mitani et al., 2009a and in some dicotyledons, cucumber, pumpkin, and soybean (Deshmukh et al., 2013) for example (see Deshmukh et al., 2015; Deshmukh and Bélanger, 2016 for others), it is likely that *F. arundinacea* has Si transporters and that differences in these underlie differences in Si uptake and deposition we observe between varieties. Other studies have found intraspecific differences in uptake abilities in rice (Wu et al., 2006; Ma et al., 2007) which revealed that the higher Si accumulating genotypes were able to accumulate more Si due to a higher level upregulation of Si transporters. Perhaps this is also the case for the differences in these varieties and may also be why the high accumulating variety (H) is better able to respond to damage as there is a greater number of Si transporters present. The spacing between the conserved (asparagine-proline-alanine (NPA)) domains in Si transporters is also likely to influence uptake abilities within and between species; the spacing between these amino acids have been shown to determine whether plants are able to import or reject importing Si into the root cells (Deshmukh et al., 2015).

## Conclusion

Few studies have looked at the relationship between Si accumulation and transpiration, and to date none have looked at these in combination with damage. To date, no studies have looked at differential expression of the Si transporters between undamaged and damaged conditions to test for molecular evidence of Si-induced responses. There were clear differences in the response of the three varieties to the damage treatments within this study, suggesting that damage is an important driver in the accumulation of Si. Removal of differences in stomatal conductance also removed the difference in Si levels between the varieties, suggesting that transpiration has a role in Si accumulation, but the higher Si levels under damaged, bagged conditions show these increases must occur by mechanisms other than just passive movement of Si in the transpiration stream. This gives clear evidence for active Si-induced defenses within this species. Further, we provide the first evidence of molecular based Si-induced defenses by the up-regulation of the active Si transporter, *Lsi2*, in damaged plants. Clearly, further molecular characterization of the mechanisms involved in Si uptake and transport following damage is necessary to fully understand how Si gets from the xylem and into the cells in the leaves. These results not only provide evidence for Si-defenses being regulated at gene level, they also provide insights into target traits for selecting plant genotypes resistant to herbivory for agriculture and other uses.

## Author Contributions

All authors designed the research. EM conducted the experiments, data analysis, and wrote the manuscript. IL advised on RNA-Seq work. SH and SM-M revised the manuscript. All authors read and approved the manuscript.

## Conflict of Interest Statement

The authors declare that the research was conducted in the absence of any commercial or financial relationships that could be construed as a potential conflict of interest.

## Footnotes

1 https://github.com/marcelm/cutadapt

2 https://github.com/trinityrnaseq/trinityrnaseq/wiki

3 http://www.cbs.dtu.dk/services/TMHMM/
