# Supplementary material for: Evidence for Active Uptake and Deposition of Si-based Defenses in Tall Fescue
Source: Front Plant Sci. 2017 Jul 18;8:1199. doi: 10.3389/fpls.2017.01199 (PMC5513917; doi:10.3389/fpls.2017.01199)
Supplement: Supplementary file 1 [file Table_1.DOCX]

Supplementary Material

Evidence for active uptake and deposition of Si based defenses in Tall Fescue

Emma McLarnon, Simon McQueen-Mason, Ingo Lenk, Susan E Hartley*

*** Correspondence:** Corresponding Author: sue.hartley@york.ac.uk

# Supplementary Data

***Table S1: Isoform sequences and percentage identity to known Lsi2 transporter sequences. N.B. ArsB is a group of efflux transporter proteins and Lsi2 belongs to this group.***

***Table S2: Trichome and phytolith density of plants under unbagged condition. VVS=very very soft, VS=very soft and H=harsh***

| UNBAGGED | | | | | | |
| --- | --- | --- | --- | --- | --- | --- |
| Treatment | Trichomes per mm^2^ | | | Phytoliths per mm^2^ | | |
|  | VVS | VS | H | VVS | VS | H |
| Undamaged plant | 3 | 6 | 6 | 16 | 21 | 15 |
| Undamaged leaves, damaged plant | 3 | 5 | 6 | 14 | 23 | 13 |
| Damaged leaves, damaged plant | 3 | 4 | 5 | 13 | 23 | 13 |

***Table S3: Trichome and phytolith density of plants under bagged conditions. VVS=very very soft, VS=very soft and H=harsh***

| BAGGED | | | | | | |
| --- | --- | --- | --- | --- | --- | --- |
| Treatment | Trichomes per mm^2^ | | | Phytoliths per mm^2^ | | |
|  | VVS | VS | H | VVS | VS | H |
| Undamaged plant | 1 | 4 | 5 | 16 | 25 | 13 |
| Undamaged leaves, damaged plant | 1 | 4 | 4 | 13 | 19 | 10 |
| Damaged leaves, damaged plant | 1 | 4 | 3 | 16 | 18 | 11 |
